# Associations between retinal microvasculature and cognition in middle-aged adults with type 1 diabetes without overt neurological symptoms

**DOI:** 10.1016/j.cccb.2026.100536

**Published:** 2026-03-16

**Authors:** Iiris Kyläheiko, Aleksi Tarkkonen, Linda Kuusela, Juha Martola, Teemu I. Paajanen, Jussi Virkkala, Ward Fickweiler, George L. King, Jennifer K. Sun, Joni A. Turunen, Marika I. Eriksson, Per-Henrik Groop, Lena M. Thorn, Turgut Tatlisumak, Jukka Putaala, Hanna Jokinen, Daniel Gordin, FinnDiane Study Group

**Affiliations:** a Department of Psychology, Faculty of Medicine, University of Helsinki, University of Helsinki, PO Box 21 (Haartmaninkatu 3) 00014 University of Helsinki, Helsinki, Finland; b Department of Neurology, Kymenlaakso Central Hospital, Kotkantie 41, 48210 Kotka, Finland; c Minerva Foundation Institute for Medical Research, Biomedicum Helsinki 2U, Tukholmankatu 8, 00290 Helsinki, Finland; d Folkhälsan Research Center, Biomedicum Helsinki, Haartmaninkatu 8, 00290 Helsinki, Finland; e HUS Medical Imaging Center, Department of Radiology, Helsinki University Hospital and University of Helsinki, Haartmaninkatu 4, 00029 HUS, Finland; f Research Program for Clinical and Molecular Metabolism, University of Helsinki, Biomedicum Helsinki, Haartmaninkatu 8, 00290 Helsinki, Finland; g Occupational Health Unit, Finnish Institute of Occupational Health, Topeliuksenkatu 41 b, 00250 Helsinki, Finland; h Department of Clinical Neurophysiology, HUS Diagnostic Center, Helsinki University Hospital and University of Helsinki, Haartmaninkatu 4, 00029 HUS, Finland; i Joslin Diabetes Center, Harvard Medical School, 1 Joslin Place, Boston, MA 02215, USA; j Department of Ophthalmology, Harvard Medical School, 243 Charles Street, Boston, MA 02114, USA; k Department of Medicine, Harvard Medical School, 25 Shattuck Street, Boston, MA 02115, USA; l Department of Ophthalmology, University of Helsinki and Helsinki University Hospital, PO Box 22 (Haartmaninkatu 4) 00014 University of Helsinki, Helsinki, Finland; m Department of Nephrology, Helsinki University Hospital and University of Helsinki, Biomedicum 2 Helsinki, Tukholmankatu 8, 00029 HUS, Finland; n Department of Diabetes, Central Clinical School, Monash University, Alfred Centre, 99 Commercial Road, Melbourne, VIC 3004, Australia; o Baker Heart and Diabetes Institute, Melbourne, VIC 3004, Australia; p Department of General Practice and Primary Health Care, Helsinki University Hospital and University of Helsinki, Tukholmankatu 8 B, 00290 Helsinki, Finland; q Department of Clinical Neuroscience, Institute of Neuroscience and Physiology, Sahlgrenska Academy at the University of Gothenburg, Blå stråket 7, plan 3, Sahlgrenska 41345 Gothenburg, Sweden; r Department of Neurology, Sahlgrenska University Hospital, Blå stråket 5, 41345 Gothenburg, Sweden; s Department of Neurology, Helsinki University Hospital and University of Helsinki, Haartmaninkatu 4, 00029 HUS, Finland; t Division of Neuropsychology, HUS Neurocenter, Helsinki University Hospital and University of Helsinki, PO box 302 (Paciuksenkatu 21), 00029 HUS, Finland; u Helsinki Hypertension Center of Excellence, University of Helsinki and Helsinki University Hospital, Haartmaninkatu 8, 00029 HUS, Finland

**Keywords:** Cognition, Cerebral small vessel disease, Optical coherence tomography angiography, Retina, Type 1 diabetes

## Abstract

**Aims:**

Type 1 diabetes is a risk factor for retinal microvascular pathology, cerebral small vessel disease (cSVD), and cognitive deficits. In older individuals with type 1 diabetes, decreased vessel densities of superficial (SCP) and deep (DCP) retinal capillary plexuses have been associated with cognitive impairment. We assessed whether these associations are already evident in midlife among individuals without overt neurological symptoms. We also investigated whether cerebral microbleeds (CMBs) and white matter hyperintensities (WMHs), manifestations of cSVD, modify these associations.

**Methods:**

We measured vessel density of the SCP and DCP, and area of foveal avascular zone (FAZ) in macular 3×3 mm optical coherence tomography angiography (OCTA) images and performed a comprehensive neuropsychological assessment and brain MRI in 153 adults with type 1 diabetes (age 46.4±7.5 years, diabetes duration 30.7±10.1 years).

**Results:**

In univariate analyses, SCP, DCP, and FAZ were related to measures of processing speed and executive functions (standardized *β* ranged from –0.29 to –0.19, *p*-values from <0.001 to 0.018). However, after adjusting for age and relevant clinical markers, only association between FAZ and a sensitive working memory task (standardized *β*=–0.25, *p*<0.05) remained significant. In interaction analyses, CMBs and WMHs did not modify these associations.

**Conclusions:**

Enlarged FAZ was associated with poorer working memory performance independently of confounding factors. Otherwise, age largely explained the associations between retinal microvascular structure and cognition in middle-aged individuals with type 1 diabetes without any overt neurological symptoms.

## Highlights

• In middle-aged individuals with type 1 diabetes (T1D), cerebral small vessel disease (cSVD) relates to poorer cognition and retinal microvascular pathology on OCTA.
• Whether early retinal microvascular changes associate with cognition in this population remains uncertain.
• Larger foveal avascular zone (FAZ) was associated with poorer working memory in middle-aged individuals with T1D.

## 1 Introduction

Type 1 diabetes poses a significant risk for cognitive impairment already in middle age, particularly in the domains of psychomotor speed and executive functions [1,2]. Executive functions refer to a set of skills supporting complex goal-directed behavior including cognitive flexibility, inhibitory control, and working memory [3]. One contributing factor for the cognitive impairment is cerebral small vessel disease (cSVD), which typically in older age manifests on brain imaging as recent small subcortical infarcts, lacunes, white matter hyperintensities (WMHs), perivascular spaces, cerebral microbleeds (CMBs), cortical superficial siderosis, brain atrophy, and cortical cerebral microinfarcts [4,5]. In our study on middle-aged individuals with type 1 diabetes, the most prominent cSVD features were CMBs and mild WMHs [6,7]. Moreover, our recent study showed that CMBs were associated with slower processing speed and impaired executive functions in middle-aged adults with type 1 diabetes without any evident neurological signs or symptoms, while mild WMHs (Fazekas score 1 in most cases) had no significance for cognitive performance [8].

The retina and brain both derive from ectoderm and thus are part of the central nervous system and share similar microvasculature [9]. Furthermore, cSVD, particularly CMBs, associates with the severity of diabetic retinopathy in middle-aged individuals with type 1 diabetes in our cohort [10]. However, prior to the development of clinically evident diabetic retinopathy, young adults (mean age 22–36 years) with type 1 diabetes may already manifest subtle microvascular retinal abnormalities in optical coherence tomography angiography (OCTA) images [[11], [12], [13], [14]]. OCTA is a novel noninvasive imaging tool providing high-resolution images of the retinal vasculature [15,16]. Indeed, our preliminary findings show that middle-aged individuals with type 1 diabetes and CMBs have significantly smaller vessel density of SCP and DCP compared to those with type 1 diabetes and no CMBs [17].

Retinal imaging has emerged as a potential cost-effective indicator of brain health [18] and cSVD [19]. Furthermore, retinal microvascular abnormalities assessed with OCTA have been associated with cSVD-related cognitive decline [20,21] among older people (mean age 63–75 years), although not consistently [22], as well as in older individuals with type 2 diabetes (mean age 65 years) [23]. Nevertheless, in older individuals with type 2 diabetes and mild cognitive impairment, but no clinically evident diabetic retinopathy, results on OCTA parameters and cognitive performance have been mixed [24,25]. An earlier study showed decreased retinal capillary vessel density to associate with worse memory and psychomotor speed in on average 63-year-old individuals with long duration of type 1 diabetes [26]. However, in younger middle-aged adults with type 1 diabetes, the associations between OCTA markers and cognition have not been studied. Therefore, we aimed to assess whether retinal microvascular changes measured by OCTA are associated with processing speed and executive functions in middle-aged individuals with type 1 diabetes. Additionally, for the first time in this population, we assessed whether CMBs, WMHs, and microvascular retinal markers have a combined effect on cognition.

## 2 Materials and methods

### 2.1 Participants and study protocol

This is a sub-study [6] of the longitudinal Finnish Diabetic Nephropathy Study (FinnDiane Study; Supplemental Table 1). The baseline measurements were conducted in 2011–2017 and the primary inclusion criteria were age of 18–50 years at enrolment, diabetes onset age of <40 years, and no kidney replacement therapy, history of overt neurovascular disease, assessed with the Questionnaire for Verifying Stroke-Free Status [27] and review of medical records, or contraindications for brain MRI [6]. During 2019–2024, we performed follow-up clinical examinations and brain MRI [7], with the study protocol extended by OCTA [17] and neuropsychological assessment [8]. The current study assesses cross-sectional associations from this second phase of the sub-study. The recruitment of study participants and the exclusion criteria for the current analyses are described in Fig. 2. Exclusion criteria included neurological diseases affecting the brain and potentially cognition (traumatic brain injury, n=3; multiple sclerosis, n=2; infarct, n=2). Those individuals from the baseline that were not included in the current OCTA subsample, did not differ at baseline in age, sex, study years, diabetes duration or HbA_1c_, as assessed with the Wilcoxon/chi-squared test (*p*>0.05). The study was approved by the ethics committee of Helsinki University Hospital and conducted according to the Declaration of Helsinki. All participants provided written informed consent.

### 2.2 Clinical examinations

At the FinnDiane Research Unit at Helsinki University Hospital, medical history, current medication, office blood pressure, waist circumference, and body mass index were evaluated (Table 1). Glycated hemoglobin (HbA_1c_), lipids, and creatinine were measured from blood samples. The estimated glomerular filtration rate (eGFR) was calculated using the Chronic Kidney Disease Epidemiology Collaboration 2009 equation. Albumin excretion rate was classified based on two out of three consecutive urine samples following standard criteria. Demographic and lifestyle data were gathered with questionnaires [6].

**Table 1:** Demographic and clinical characteristics of the participants with type 1 diabetes (T1D).

	Participants (n=153)
Age, years	46.4 (7.5)
Female sex, n (%)	79 (51.6)
Education, years	16.8 (3.4)
Age at diagnosis of T1D, years	16.4 (9.4)
T1D duration, years	30.7 (10.1)
BMI, kg/m^2^	28.2 (5.1)
Waist-to-height ratio	0.55 (0.08)
Systolic blood pressure, mmHg	130 (16)
Diastolic blood pressure, mmHg	82 (9)
Antihypertensive medication, n (%)	71 (46.4)
HbA_1c_, mmol/mol	59 (10)
HbA_1c_, %	7.6 (0.9)
Total cholesterol, mmol/L	4.1 (1.0)
LDL cholesterol, mmol/L	2.3 (0.9)
HDL cholesterol, mmol/L	1.4 (0.4)
Triglycerides, mmol/L	1.1 (0.6)
Coronary heart disease, n (%)	3 (2.0)
Current smoker, n (%)	7 (4.6)
Former smoker, n (%)	45 (29.6)
Moderately or severely increased albuminuria, n (%)	32 (20.9)
Estimated glomerular filtration rate, ml/min/1.73m^2^	102 (15)
Retinal photocoagulation, n (%)	42 (27.5)
**Retinal optical coherence tomography angiography markers**	
Density of SCP, %	45.41 (3.83)
Density of DCP, %	51.04 (3.60)
FAZ, mm^2^	0.31 (0.14)
**Brain magnetic resonance imaging markers**	
CMB (≥1), n (%)	53 (34.9)
WMHs	
No WMHs (Fazekas grade 0), n (%)	46 (30.3)
Fazekas mild (grade 1), n (%)	104 (68.4)
Fazekas moderate (grade 2), n (%)	2 (1.3)
WMH volume, cm^3^	1.26 (1.72)
Lacunar infarcts, n (%)	3 (2.0)

Data are presented as mean (SD), if not mentioned otherwise. BMI, body mass index; CMB, cerebral microbleed; DCP, deep retinal capillary plexus; FAZ, foveal avascular zone; HbA_1c_, glycated hemoglobin; HDL, high-density lipoprotein; LDL, low-density lipoprotein; SCP, superficial retinal capillary plexus; T1D, type 1 diabetes; WMH, white matter hyperintensity.

### 2.3 Optical coherence tomography angiography (OCTA)

**Fig. 1:**
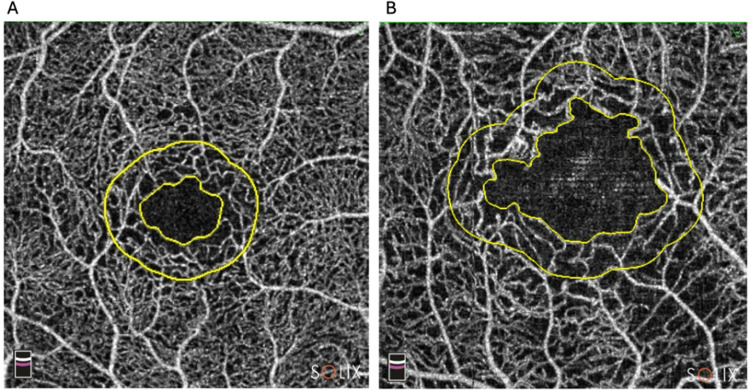
Optical coherence tomography angiography images of A) normal and B) enlarged foveal avascular zone (individual with 83 cerebral microbleeds) marked with the inner yellow circle.

OCTA imaging was performed at Helsinki Biomedicum Research Center during the clinical examination. OCTA images were obtained with the SOLIX 1.0 Optovue (Optovue Inc, Fremont, CA, USA) with signal strength of 55 or more, with a lateral resolution of 15 μm, axial resolution of 5 μm, using AngioVue® 3 × 3 mm protocol. Both eyes were scanned in a 3 × 3 mm region centered on the fovea. Vessel density of SCP and DCP, and area of FAZ (Fig. 1) were measured with automated software using Projection Artifact Removal (3D PAR). The software was applied using the manufacturer’s default settings; no additional customization was performed.

**Fig. 2:**
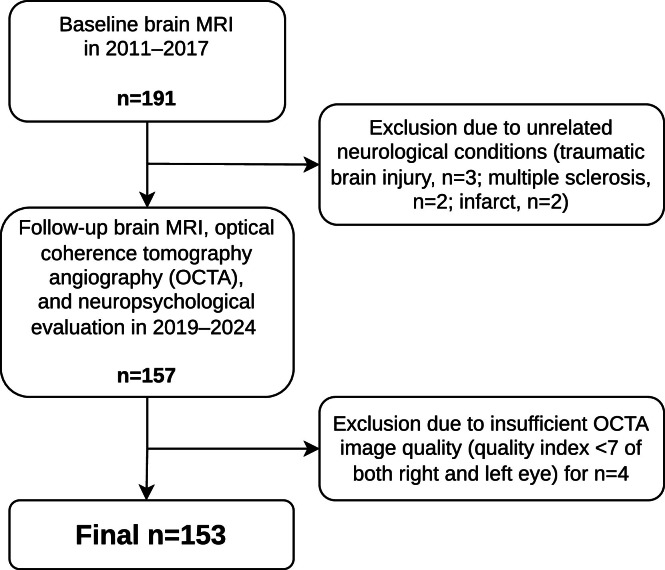
Flowchart of the participant recruitment and data acquisition for the current study.

For the analyses, the right eye of the participant was selected, unless the image quality was insufficient, defined as <7. In this case, the left eye was selected. If both eyes were of inadequate quality, they were discarded from the analyses (Fig. 2).

### 2.4 Brain MRI

Brain MRI (n=152) was conducted at Helsinki University Hospital's Medical Imaging Center with 3T Philips Ingenia (Best, The Netherlands) and a 32-channel head coil [7]. MRI sequences included T1, T2, FLAIR, susceptibility-weighted imaging, T2*, diffusion-weighted imaging, T1 Magnetization-Prepared Rapid Gradient Echo, and MR time-of-flight. Senior neuroradiologist (JM) assessed visually the number of CMBs, severity of WMHs by Fazekas scale, and infarcts and lacunes according to standard criteria [4,28], blinded to clinical data, OCTA, and neuropsychological evaluation.

WMHs were segmented from 3D FLAIR images using the Lesion Prediction Algorithm [29], integrated into the Lesion Segmentation Tool for SPM (version 3.0.0; www.statistical-modelling.de/lst.html) (*n*=146). To correct for individual differences in brain size, WMH volumes were normalized by dividing them by the total intracranial volume [30], which was obtained through segmentation of the T1 and T2 3D TSE sequence Freesurfer (version 7.1.1) segmentation [31,32]. Any required adjustments to the segmentations were made manually by a skilled physicist (LK).

### 2.5 Neuropsychological assessment

Extensive neuropsychological assessment [8] was performed by trained research psychologists. The current analysis included measures of processing speed and executive functions as the core cognitive domains affected in type 1 diabetes [1]. These methods are described in detail in Supplemental Table 2. Processing speed was evaluated with Wechsler Adult Intelligence Scale IV (WAIS-IV) Coding [33], Stroop Color-Naming (Stroop-II) [34], and the computerized Flexible Attention Test (FAT) [35] subtasks of Reaction Time (FAT-RT) and Numbers (FAT-N). FAT is a computerized tablet test, based on the widely-used Trail making test and Corsi block tapping test [36,37], including eight tasks of progressing difficulty covering the domains of visuomotor speed, set shifting, and working memory, and has been shown to correlate with corresponding traditional neuropsychological tests [35,38]. An illustration of FAT can be found in an earlier study [38]. Cognitive flexibility was assessed with FAT subtests of Numbers and Letters (FAT-NL), Numbers and Shapes (FAT-NS), and Numbers and Months Forward (FAT-NMF) and Backward (FAT-NMB) [35]. Inhibitory control was assessed with Stroop Color-Incongruent (Stroop-III) [34]. Working memory was measured with FAT Visuospatial Memory Span Forward (FAT-MF) and Backward (FAT-MB) [35,38]. Participants were instructed to avoid hypoglycemic events 24h preceding the assessment. Glucose level was checked either using capillary test or with participants own glucose monitoring sensor, if available before the examination and required to be ≥3 mmol/L.

### 2.6 Statistical analysis

Spearman correlation was used to assess the association between glucose level prior to the neuropsychological assessment and cognitive performance. We report the data as mean (standard deviation, sd), if not specified otherwise, and the results as standardized beta coefficients.

A few missing values existed in cognitive test scores due to the participant’s failure to complete the task or technical reasons. The number of missing values ranged from 1 to 2 in all test scores, except for FAT-MB, which had 15 missing values. Assessed with t-test and Wilcoxon test, where appropriate, age, sex, years of education, WMH volumes, or CMBs did not differ between individuals with missing values in FAT-MB and those with available data. Missing data were not imputed.

To examine the cross-sectional associations and modifying factors between retinal markers and cognition, we performed linear regression analyses hierarchically with cognitive test scores as dependent variables. To facilitate comparability of regression coefficients across predictors, all continuous variables were z-standardized (mean=0, SD=1) prior to inclusion in the models. First, we assessed the univariate associations of the vessel densities of SCP and DCP, and FAZ entered as independent variables, one-by-one. Subsequently, age was included in the model as the main confounder (simultaneous entry method). As sensitivity analyses, we first assessed the effect of potential confounding clinical factors, i.e., eGFR, systolic blood pressure, and HbA_1c_ [8], on the models. In the second set of sensitivity analyses, we added age, years of education, self-reported history of depression (yes/no), and smoking status (current or former smoker vs never smoked) as independent variables in the model. Finally, interaction terms (OCTA marker * MRI marker) were included into the multivariable model with age to assess the combined effects of OCTA and cSVD markers (CMB number and continuous WMH volume, one at a time). For the interaction analysis, participants were categorized by their CMB number (0, 1-2, ≥3; *n*=99, *n*=30, and *n*=23, respectively), with 0 CMBs as a reference category [8].

Dependent variables were logarithmically transformed, if appropriate. No systematic evidence for violation of homoscedasticity was found visually. Approximately one third of the models showed a slight violation of the residual normality assumption, partly due to outliers. Cook’s distance for influential data points was nevertheless consistently below 1. All analyses were run additionally without influential data points, and this did not affect those results that were significant after false discovery rate (FDR) correction.

Uncorrected *p*-values were examined for descriptive purposes using a threshold of *p*<0.05 for all linear regression models. To control for multiplicity, FDR correction was subsequently applied across all models using the Benjamini–Hochberg procedure. FDR-corrected *p*-values <0.05 were considered statistically significant.

Effect sizes for the significant associations between OCTA markers and cognition in linear regression models were reported with Cohen’s *f^2^.* Statistical analyses were performed with R version 4.5.2.

## 3 Results

### 3.1 Participant characteristics

For the analyses, 153 participants with type 1 diabetes were available (Fig. 2). Descriptive data of participants are presented in Table 1. Glucose levels prior to the neuropsychological evaluation were in 87% between 4.0–13.9 mmol/L, in 10% ≥14 mmol/L, and in 3% 3.0–3.9 mmol/L. The glucose level did not correlate with performance in any of the cognitive tasks (Spearman ρ ranging from –0.02 to 0.15**,** *p*>0.05). Of the participants, 21.6% (*n*=33) reported history of depression. Median time interval between OCTA measurement and neuropsychological assessment was 17.0 days (IQR 6.0–35.0) and between MRI and neuropsychological evaluation 14.0 days (IQR 5.0–27.0).

### 3.2 Univariate associations between OCTA markers and cognition

In univariate models, SCP was related to processing speed (Coding, Stroop-II, FAT-RT, and FAT-N) and executive function subdomains of inhibitory control (Stroop-III) and cognitive flexibility (FAT-NL, FAT-NMF, and FAT-NMB) (standardized *β*=–0.26–0.17, *p*-values ranged from 0.001 to 0.042, Cohen’s *f^2^*=0.03–0.07) (for details, see Supplemental Table 3). DCP was associated with processing speed (Coding, Stroop-II, and FAT-RT) and executive function subdomains of inhibitory control (Stroop-III) and cognitive flexibility (FAT-NL and FAT-NMF) (standardized *β*=–0.028–(–0.17), *p*-values ranged from <0.001 to 0.036, Cohen’s *f^2^*=0.03–0.09). FAZ was linked to executive function measures of cognitive flexibility (FAT-NL) and working memory (FAT-MB) (standardized *β*=–0.26–0.17, *p*-values ranged from <0.001 to 0.034, Cohen’s *f^2^*=0.03–0.09).

### 3.3 Multivariate associations between OCTA markers and cognition

**Fig. 3:**
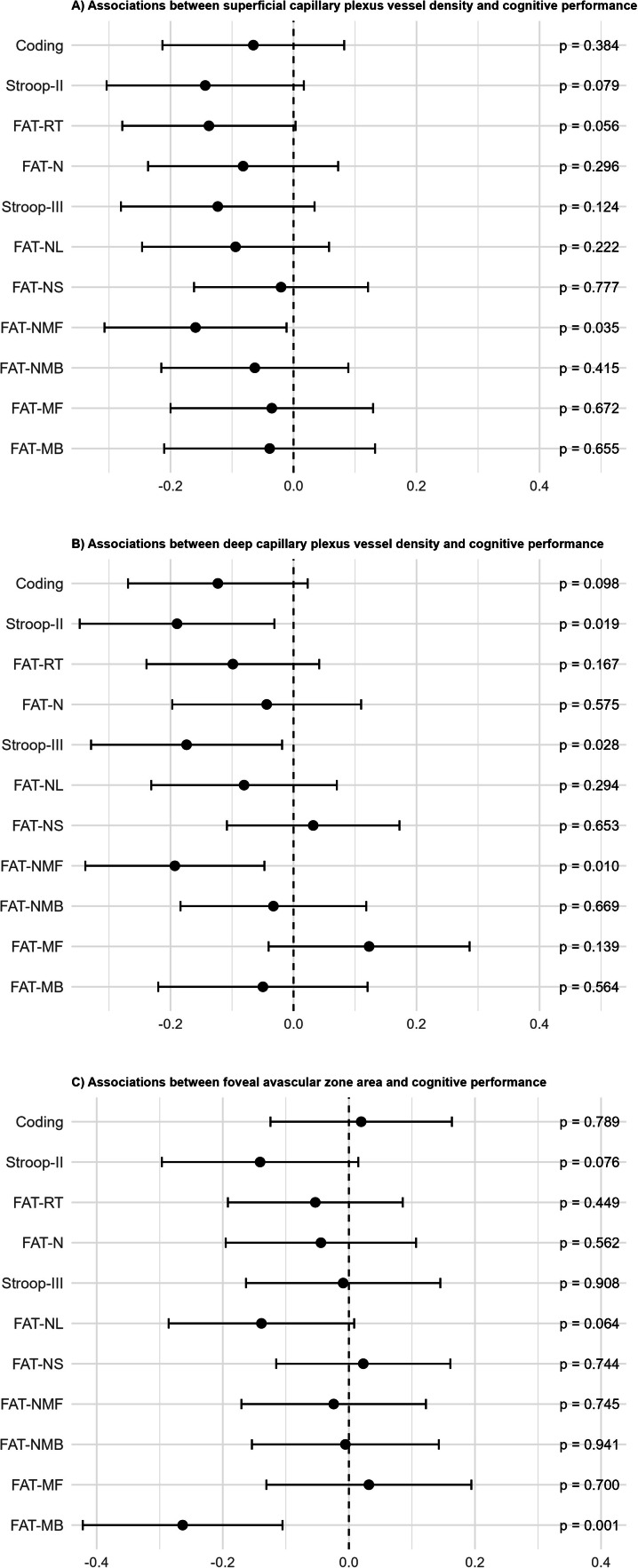
Forest plots showing age-corrected associations between optical coherence tomography angiography markers and cognition. Values are standardized *β* coefficients with 95% confidence intervals derived from linear regression models and *p*-values. In panels A and B, lower vessel density reflects poorer microvascular health and is associated with worse cognitive performance. Because larger foveal avascular zone (FAZ) values indicate greater microvascular pathology, FAZ associations were sign-reversed to harmonize effect directions so that lower values consistently represent worse retinal microvascular status. In addition, associations for Coding and the Flexible Attention Test (FAT) visuospatial memory span subtasks (Forward [MF] and Backward [MB]) were sign-reversed to ensure consistent interpretation across cognitive measures. FAT-N, FAT Numbers; FAT-NL, FAT Numbers and Letters; FAT-NMB, FAT Numbers and Months Backward; FAT-NMF, FAT Numbers and Months Forward; FAT-NS, FAT Numbers and Shapes; FAT-RT, FAT Reaction Time; Stroop-II, Stroop Color-Naming; Stroop-III, Stroop Color-Incongruent. Illustration of FAT can be found in an earlier study [38].

After adjusting for age as the main confounder (Table 2 and Fig. 3), the associations between SCP and the cognitive flexibility measure FAT-NMF remained significant (Cohen’s *f^2^*=0.03). Instead, SCP and processing speed (Coding, Stroop-II, FAT-RT, and FAT-N), inhibitory control (Stroop-III), and most of the cognitive flexibility (FAT-NL and FAT-NMF) measures were not significantly related. Also, relations between DCP and processing speed (Stroop-II) and executive function measures of inhibitory control (Stroop-III) and cognitive flexibility (FAT-NMF) (Cohen’s *f^2^*=0.03–0.05) remained significant. Instead, associations between DCP and some of the processing speed (Coding and FAT-RT) and executive cognitive flexibility measures (FAT-NL) did not remain significant. Likewise, the association for FAZ and working memory (FAT-MB) remained significant (Cohen’s *f^2^*=0.08), whereas the association between FAZ and cognitive flexibility (FAT-NL) did not. R² and R² change for age-adjusted models are reported in Supplemental Table 4.

**Table 2:** Cross-sectional age-corrected associations of vascular density for superficial (SCP) and deep (DCP) retinal capillary plexus, and foveal avascular zone (FAZ) with cognitive tests of processing speed and executive function subdomains of inhibition, cognitive flexibility, and working memory.

	Processing Speed				Executive Functions						
					Inhibition	Cognitive flexibility				Working memory	
	Coding	Stroop-II	FAT-RT	FAT-N	Stroop-III	FAT-NL	FAT-NS	FAT-NMF	FAT-NMB	FAT-MF	FAT-MB
	Standardized *β* (*p*-value)										
SCP	0.07 (0.384)	-0.14 (0.079)	-0.14 (0.056)	-0.08 (0.296)	-0.12 (0.124)	-0.09 (0.222)	-0.02 (0.777)	**-0.16** **(0.035)**	-0.06 (0.415)	0.04 (0.672)	0.04 (0.655)
Age	**-0.44** **(<0.001)**	**0.20** **(0.017)**	**0.49** **(<0.001)**	**0.35** **(<0.001)**	**0.27** **(<0.001)**	**0.38** **(<0.001)**	**0.53** **(<0.001)**	**0.40** **(<0.001)**	**0.40** **(<0.001)**	**-0.19** **(0.025)**	-0.17 (0.054)
DCP	0.12 (0.098)	**-0.19** **(0.019)**	-0.10 (0.167)	-0.04 (0.575)	**-0.17** **(0.028)**	-0.08 (0.294)	0.03 (0.653)	**-0.19** **(0.010)**	-0.03 (0.669)	-0.12 (0.139)	0.05 (0.564)
Age	**-0.43** **(<0.001)**	**0.19** **(0.020)**	**0.50** **(<0.001)**	**0.36** **(<0.001)**	**0.26** **(<0.001)**	**0.39** **(<0.001)**	**0.54** **(<0.001)**	**0.40** **(<0.001)**	**0.41** **(<0.001)**	**-0.23** **(0.007)**	-0.17 (0.060)
FAZ	0.02 (0.789)	0.14 (0.076)	0.05 (0.449)	0.04 (0.562)	0.01 (0.908)	0.14 (0.064)	-0.02 (0.744)	0.02 (0.745)	0.01 (0.941)	0.03 (0.700)	**-0.27** **(0.001)^†^**
Age	**-0.46** **(<0.001)**	**0.22** **(0.006)**	**0.52** **(<0.001)**	**0.37** **(<0.001)**	**0.30** **(<0.001)**	**0.40** **(<0.001)**	**0.54** **(<0.001)**	**0.44** **(<0.001)**	**0.42** **(<0.001)**	**-0.20** **(0.014)**	-0.15 (0.069)

Multivariable linear regression models with cognitive test scores as dependent variables. Independent variables included separately vascular density of SCP, vascular density of DCP, and FAZ and additionally age as the main confounder. Values are standardized beta coefficients (*p*-values). FAT, Flexible Attention Test; FAT-MB, FAT Visuospatial Memory Span Backward; FAT-MF, FAT Visuospatial Memory Span Forward; FAT-N, FAT Numbers; FAT-NL, FAT Numbers and Letters; FAT-NMB, FAT Numbers and Months Backward; FAT-NMF, FAT Numbers and Months Forward; FAT-NS, FAT Numbers and Shapes; FAT-RT, FAT Reaction Time; Stroop-II, Stroop Color-Naming; Stroop-III, Stroop Color-Incongruent. Associations between optical coherence tomography angiography (OCTA) markers and cognition that remain significant after false discovery rate correction are marked with **†**. The independent contribution of OCTA markers on cognition was small (Cohen’s *f^2^* for significant associations ranged between 0.03 and 0.08). Illustration of FAT can be found in an earlier study [38].

In the first set of sensitivity analyses, after controlling for the main clinical characteristics, i.e., eGFR, systolic blood pressure, and HbA_1c_, the significant associations between OCTA markers and cognitive measures mainly remained same, only one association between SCP and Stroop-II became additionally significant (Supplemental Tables 5, 6, and 7; Cohen’s*f^2^* for SCP=0.03–0.04, DCP=0.02–0.05, and FAZ=0.07). The results were same in the second set of sensitivity analyses, when education, depression, and smoking history were included in the model with age (Supplemental Tables 8, 9, and 10; Cohen’s *f^2^* for SCP=0.03, DCP=0.04–0.05, and FAZ<0.01).

### 3.4 FDR correction for multiple testing

After correcting for multiplicity, significant univariate associations for SCP and processing speed (Stroop-II [FDR-corrected *p*=0.040] and FAT-RT [*p*=0.006]) and executive functions (Stroop-III [*p*=0.040], FAT-NL [*p*=0.040], and FAT-NMF [*p*=0.006]) remained significant. Also, univariate relations between DCP and processing speed (Coding [*p*=0.017], Stroop-II [*p*=0.015], and FAT-RT [*p*=0.018]) and executive functions (Stroop-III [*p*=0.015] and FAT-NMF [*p*=0.004]) remained significant. Univariate relation between FAZ and working memory (FAT-MB) stayed significant (*p*=0.008) as well.

Significant associations in multivariable models adjusted for age assessing SCP and DCP did not remain after FDR correction, but for FAZ and working memory (FAT-MB) the significance persisted (FDR-corrected *p*=0.011; Table 2). Similarly in the sensitivity analyses adjusting for clinical factors, the significant association between FAZ and FAT-MB remained significant after FDR correction (FDR-corrected *p*=0.033; Supplemental Table 7). In the second set of sensitivity analyses adjusting for education, depression, and smoking, associations did not remain significant after FDR-correction; however, the standardized beta coefficients were of comparable magnitude to those observed in models adjusted for age and additionally clinical factors.

### 3.5 Interaction effect of selected cSVD markers on associations between OCTA markers and cognition

An interaction between vessel density of DCP (%) and the group with ≥3 CMBs for Stroop-II (standardized *β*=–0.45, *p*=0.039) was observed, indicating a synergistic effect between a higher number of CMBs and smaller vessel density of DCP on slower processing speed. A relation between FAZ area (mm²) and log-transformed WMH volume for a processing speed measure of Coding (standardized *β*=–0.20, *p*=0.026) was observed. Other MRI marker * OCTA marker interactions were non-significant (data not shown). However, neither of these interactions remained significant after FDR correction performed separately for interaction models over CMBs and WMH volumes.

## 4 Discussion

This study investigated associations between retinal microvasculature measured by OCTA and cognition among middle-aged individuals (mean age 46 years) with type 1 diabetes without any overt neurological symptoms. Our main finding was that larger FAZ, a marker of microvascular retinal pathology, was related to poorer working memory performance, independently of age and the main type 1 diabetes–related clinical markers. As expected, age largely explained the associations found between decreased vessel densities of DCP and SCP and poorer processing speed and executive function subdomains of inhibitory control and cognitive flexibility. The number of CMBs or WMH volume did not modify the associations between the vessel density of DCP and SCP or FAZ area, and cognitive performance.

To our knowledge, no earlier study has examined whether decreased vascular density of SCP and DCP is related to cognition in individuals with type 1 diabetes in middle age, or whether enlarged FAZ is linked with cognition in type 1 diabetes in general. In our study, we included individuals without any symptoms of neurological disorders to identify associations between incipient microvascular retinal changes and cognition. We found evidence for a link between enlarged FAZ and weakened working memory. In an earlier study, in 63-year-old individuals with long duration of type 1 diabetes [26], a decreased vessel density of SCP was linked with poorer psychomotor speed, and decreased vessel density in DCP with worse delayed memory. We found no such evidence in the models corrected for age and clinical or other central confounding factors. However, the earlier study differed from this study in several methodological aspects, such as inclusion of younger participants with shorter diabetes duration, a larger sample size, and the correction for multiple testing.

There are also a few studies on OCTA and cognition in type 2 diabetes, and the results are partly inconsistent. An earlier study observed a decreased SCP vessel density in those with type 2 diabetes (mean age 73 years) and mild cognitive impairment (MCI) compared to those with mild cognitive impairment but no diabetes [25]. However, they found no difference in FAZ among the groups [25]. In another study conducted in older people (mean age 65 years) with type 2 diabetes, vessel density in DCP but not in SCP was associated with one cognitive screening subscore [23]. Associations between FAZ and cognition were not examined [23]. In older individuals with type 2 diabetes (median age 74 years), an earlier study found no associations between SCP, DCP, and FAZ, and the absence or presence of MCI [24]. However, their study differed from ours in numerous ways, including study design, inclusion of older participants with shorter diabetes duration, different OCTA device, and use of neuropsychological tests [24].

Our finding that FAZ, a marker of retinal microvascular damage, was associated with working memory, adds to our earlier discovery that CMBs, marker of microvascular damage in the brain, were linked with processing speed and executive functions, i.e., inhibitory control and working memory, in middle-aged individuals with type 1 diabetes [8]. However, in the same cohort, no difference was found between FAZ areas in individuals with type 1 diabetes and with and without CMBs, even though those with CMBs had significantly smaller vessel densities of SCP and DCP compared to those without CMBs [17]. One plausible explanation might be the large variation among FAZ compared to those of DCP and SCP in our cohort [17], which may have emphasized the association. Notably, variability seems to be large also among healthy individuals [39]. Additionally, even though we found the association between FAZ and performance in a difficult visuospatial working memory test [38], the effect size for the association was small and therefore possibly not clinically relevant. We further hypothesized that those with a higher number of CMBs or larger WMH volume, as a marker of more advanced cSVD, might present stronger associations between retinal microvascular pathology and cognitive measures. Nonetheless, we did not find evidence for that, which might, however, be due to the relatively few participants with ≥3 CMBs as well as the mild severity of WMHs in the sample.

In general, type 1 diabetes is known to be a significant risk factor for accelerated brain aging and dementia [[40], [41], [42]], which may be driven by vascular mechanisms [43]. Thus, biological markers are necessary to identify those individuals with type 1 diabetes who are at risk for cognitive deficits to timely enhance the treatment of vascular risk factors. In individuals with cSVD (mean age 64 years) [20] and in individuals with type 2 diabetes–related cSVD (mean age 65 years) [23], there is already some evidence for associations between retinal OCTA markers and cognitive symptoms. However, our findings indicate that in on average 46-year-old individuals with type 1 diabetes, cognitive measures seem to be more strongly related to CMBs than OCTA markers [8].

There are some limitations to this study. As this is a cross-sectional study, we cannot draw conclusions about how retinal and cognitive changes develop over time. Additionally, the present study design limits interpretations to descriptive associations rather than underlying mechanisms between different OCTA markers and their associations with cognition. Even though the sample size of the study is relatively large compared to many earlier studies, there were significant associations that attenuated below the significance threshold after the FDR correction, which may indicate that the statistical power may have been limited. We also acknowledge that in interaction models, the CMB groups were small, which likely affects statistical power. Therefore, the results should be replicated in larger cohorts. The study sample consists of Finnish people, and a multicenter approach could have increased the generalizability of the results. We also lack data on intraocular pressure and refractive error and adjustments for these factors were not possible, even though they may influence OCTA image quality. To mitigate potential effects on image quality, we applied predefined, uniform quality criteria (signal strength ≥55) and a consistent eye-selection rule. We also did not grade diabetic retinopathy severity using the Early Treatment Diabetic Retinopathy Study (ETDRS), as our focus was on OCTA, which provides objective vascular measures beyond fundus photographs. While ETDRS grading could provide complementary information, those analyses would be better suited for a separate study. Visual acuity data were lacking, but since the visual demands are similar across all computerized tests, our findings are unlikely to be attributable to visual deficits. The strengths of this study include standardized evaluation of CMBs and WMHs, a comprehensive neuropsychological assessment of processing speed and executive functions which included traditional but also more experimental sensitive computerized measures, correction of multiple testing, and reporting of standardized effect sizes.

To conclude, this is the first study assessing associations between OCTA retinal imaging and incipient, cSVD-related cognitive changes in middle-aged individuals with type 1 diabetes and no overt neurological symptoms. Our results indicate that in this population, retinal changes are modestly associated with cognition. Longitudinal studies with larger sample sizes are warranted to investigate whether retinal microvascular changes can predict cognitive decline in clinical practice.

## Data availability

Individual-level data from the study participants are not publicly available due to consent restrictions provided by the participants at the time of data collection. Readers may propose collaboration to access the individual-level data by contacting the lead investigator.

## CRediT authorship contribution statement

**Iiris Kyläheiko:** Writing – original draft, Visualization, Validation, Methodology, Investigation, Funding acquisition, Formal analysis, Data curation, Conceptualization. **Aleksi Tarkkonen:** Writing – review & editing, Visualization, Software, Data curation, Conceptualization. **Linda Kuusela:** Writing – review & editing, Methodology, Formal analysis, Data curation. **Juha Martola:** Writing – review & editing, Methodology, Formal analysis, Data curation, Conceptualization. **Teemu I. Paajanen:** Writing – review & editing, Software. **Jussi Virkkala:** Writing – review & editing, Software. **Ward Fickweiler:** Writing – review & editing. **George L. King:** Writing – review & editing. **Jennifer K. Sun:** Writing – review & editing. **Joni A. Turunen:** Writing – review & editing, Validation, Methodology, Conceptualization. **Marika I. Eriksson:** Writing – review & editing. **Per-Henrik Groop:** Writing – review & editing, Resources, Project administration. **Lena M. Thorn:** Writing – review & editing, Resources, Project administration, Methodology, Data curation. **Turgut Tatlisumak:** Writing – review & editing, Conceptualization. **Jukka Putaala:** Writing – review & editing, Supervision, Methodology, Conceptualization. **Hanna Jokinen:** Writing – review & editing, Validation, Supervision, Resources, Project administration, Methodology, Funding acquisition, Data curation, Conceptualization. **Daniel Gordin:** Writing – review & editing, Supervision, Resources, Project administration, Methodology, Funding acquisition, Conceptualization.

## Declaration of competing interest

The authors declare the following financial interests/personal relationships which may be considered as potential competing interests:

Iiris Kylaeheiko reports financial support was provided by Kymenlaakso regional fund. Iiris Kylaeheiko reports financial support was provided by Diabetes Research Foundation (Finland). Iiris Kylaeheiko reports financial support was provided by Ministry of Social Affairs and Health. Iiris Kylaeheiko reports travel was provided by The Finnish Diabetes Association. Iiris Kylaeheiko reports travel was provided by The Finnish Neuropsychological Society. Hanna Jokinen reports financial support was provided by Research Council of Finland. Hanna Jokinen reports financial support was provided by HUS Helsinki University Hospital. Daniel Gordin reports financial support was provided by Research Council of Finland. Daniel Gordin reports financial support was provided by Minerva Foundation Institute for Medical Research. Daniel Gordin reports financial support was provided by HUS Helsinki University Hospital. Daniel Gordin reports financial support was provided by Liv och Hälsa Society. Daniel Gordin reports financial support was provided by University of Helsinki. Daniel Gordin reports financial support was provided by Medical Society of Finland (Finska Läkaresällskapet). Daniel Gordin reports financial support was provided by Sigrid Jusélius Foundation. Per-Henrik Groop reports financial support was provided by Folkhälsan Research Foundation. Per-Henrik Groop reports financial support was provided by Liv och Hälsa Society. Per-Henrik Groop reports financial support was provided by Sigrid Jusélius Foundation. Per-Henrik Groop reports financial support was provided by Wilhelm and Else Stockmann Foundation. Daniel Gordin reports a relationship with Astellas, AstraZeneca, Bayer, Boehringer Ingelheim, GE Healthcare, Novo Nordisk that includes: consulting or advisory. Daniel Gordin reports a relationship with AstraZeneca, Bayer, Boehringer Ingelheim, Fresenius, Novo Nordisk, and Ratiopharm that includes: speaking and lecture fees. Linda Kuusela reports a relationship with Astrazeneca, Orion that includes: equity or stocks. Marika Eriksson reports a relationship with Finska Läkaresällskapet that includes: funding grants. Marika Eriksson reports a relationship with BCB Medical Oy that includes: equity or stocks. Per-Henrik Groop reports a relationship with Astellas, AstraZeneca, Bayer, Berlin Chemie, Boehringer Ingelheim, Eli Lilly, Elo Water, Genzyme, Menarini, Merck Sharp & Dohme, Medscape, Novartis, Novo Nordisk, PeerVoice, Sanofi, Sciarc that includes: speaking and lecture fees. Per-Henrik Groop reports a relationship with Abbvie, Astellas, AstraZeneca, Bayer, Boehringer Ingelheim, Cebix, Eli Lilly, Janssen, Medscape, Merck Sharp & Dohme, Mundipharma, Nestle, Novartis, Novo Nordisk, Sanofi that includes: consulting or advisory. Per-Henrik Groop reports a relationship with Signe and Ane Gyllenberg Foundation, Minerva Foundation that includes: board membership. Jukka Putaala reports a relationship with Finnish Hypertension Society that includes: board membership. George King reports a relationship with NIH that includes: funding grants. George King reports a relationship with Thomas Beatson Foundation that includes: board membership. Aleksi Tarkkonen reports a relationship with BioMedicum Foundation, Finnish Society of Angiology, Helsinki University Hospital that includes: funding grants. Aleksi Tarkkonen reports a relationship with RokoteNyt Oy, Sana Biotechnologies, Terveystalo Oy, MedTronic, Novo Nordisk that includes: equity or stocks. Jennifer Sun reports a relationship with Jaeb Center, Genentech, Boehringer Ingelheim, Novo Nordisk, Roche, Physical Sciences, Inc, Breakthrough T1D, Mary Tyler Moore Vision Initiative (through Mary Tyler Moore and S. Robert Levine, MD Charitable Foundation), Massachusetts Lions Eye Research Fund that includes: funding grants. Jennifer Sun reports a relationship with Alcon, Boehringer Ingelheim, Novo Nordisk that includes: travel reimbursement. Jennifer Sun reports a relationship with Adaptive Sensory Technology, LKC Technologies, Optovue, Konan that includes: non-financial support. Hanna Jokinen is a member of CCCB editorial board. Given her role as a member of CCCB editorial board, she had no involvement in the peer review of this article and had no access to information regarding its peer review. Full responsibility for the editorial process for this article was delegated to another journal editor. If there are other authors, they declare that they have no known competing financial interests or personal relationships that could have appeared to influence the work reported in this paper.

## Appendix Supplementary materials

Image, application 1
