# Supplementary material for: Associations between retinal microvasculature and cognition in middle-aged adults with type 1 diabetes without overt neurological symptoms
Source: Cereb Circ Cogn Behav. 2026 Mar 16;10:100536. doi: 10.1016/j.cccb.2026.100536 (PMC13066947; doi:10.1016/j.cccb.2026.100536)
Supplement: Supplementary file 1 [file mmc1.pdf]

SUPPLEMENTARY MATERIAL

Supplemental Table 1. FinnDiane Study Centers and their physicians and nurses.

FinnDiane Study Centers	Physicians and nurses
**Anjalankoski Health Center**	S.Koivula, T.Uggeldahl
**Central Finland Central Hospital, Jyväskylä**	T.Forslund, A.Halonen, A.Koistinen, P.Koskiaho, M.Laukkanen, J.Saltevo, M.Tiihonen
**Central Hospital of Åland Islands, Mariehamn**	M.Forsen, H.Granlund, A.-C.Jonsson, B.Nyroos
**Central Hospital of Kanta-Häme, Hämeenlinna**	P.Kinnunen, A.Orvola, T.Salonen, A.Vähänen
**Central Hospital of Kymenlaakso, Kotka**	R.Paldanius, M.Riihelä, L.Ryysy
**Central Hospital of Länsi-Pohja, Kemi**	H.Laukkanen, P.Nyländen, A.Sademies
**Central Ostrobothnian Hospital District, Kokkola**	S.Anderson, B.Asplund, U.Byskata, P.Liedes, M.Kuusela, T.Virkkala
**City of Espoo Health Center:**	
**Espoonlahti**	A.Nikkola, E.Ritola
**Tapiola**	M.Niska, H.Saarinen
**Samaria**	E.Oukko-Ruponen, T.Virtanen
**Viherlaakso**	A.Lyytinen
**City of Helsinki Health Center:**	
**Puistola**	H.Kari, T.Simonen
**Suutarila**	A.Kaprio, J.Kärkkäinen, B.Rantaeskola
**Töölö**	P.Kääriäinen, J.Haaga, A-L.Pietiläinen
**City of Hyvinkää Health Center**	S.Klemetti, T.Nyandoto, E.Rontu, S.Satuli-Autere
**City of Vantaa Health Center:**	
**Korso**	R.Toivonen, H.Virtanen
**Länsimäki**	R.Ahonen, M.Ivaska-Suomela, A.Jauhiainen
**Martinlaakso**	M.Laine, T.Pellonpää, R.Puranen
**Myyrmäki**	A.Airas, J.Laakso, K.Rautavaara
**Rekola**	M.Erola, E.Jatkola
**Tikkurila**	R.Lönnblad, A.Malm, J.Mäkelä, E.Rautamo
**Heinola Health Center**	P.Hentunen, J.Lagerstam
**Helsinki University Hospital, Department of Medicine, Division of Nephrology**	R.Bergdal, T.Claesson, A.Dufva, N.Elonen, M.Eriksson, J.Fagerudd, M.Feodoroff, D.Gordin, P.-H.Groop, O.Heikkilä, K.Hietala, S.Hägg-Holmberg, F.Jansson Sigfrids, M.Korolainen, J.Kytö, S.Lindh, J.Nicklén, H.Paajanen, K.Pettersson-Fernholm, K.Rimpeläinen, M.Rosengård-Bärlund, M.Rönnback, L.Salovaara, A.Sandelin, M.Saraheimo, S.Satuli-Autere, R.Simonsen, P.Smidtslund, L.Thorn, H.Tikkanen, J.Tuomikangas, A.Tynjälä, K.Uljala, T.Vesisenaho, J.Wadén, A.Ylinen
**Herttoniemi Hospital, Helsinki**	V.Sipilä
**Hospital of Lounais-Häme, Forssa**	T.Kalliomäki, J.Koskelainen, R.Nikkanen, N.Savolainen, H.Sulonen, E.Valtonen
**Hyvinkää Hospital**	L. Norvio, A.Hämäläinen
**Iisalmi Hospital**	E.Toivanen
**Jokilaakso Hospital, Jämsä**	A.Parta, I.Pirttiniemi

**Jorvi Hospital, Helsinki University Central Hospital**	S.Aranko, S.Ervasti, R.Kauppinen-Mäkelin, A.Kuusisto, T.Leppälä, K.Nikkilä, L.Pekkonen
**Jyväskylä Health Center, Kyllö**	K.Nuorva, M.Tiihonen
**Kainuu Central Hospital, Kajaani**	S.Jokelainen, K.Kananen, M.Karjalainen, P.Kemppainen, A-M.Mankinen, A.Reponen, M.Sankari
**Kerava Health Center**	H.Stuckey, P.Suominen
**Kirkkonummi Health Center**	A.Lappalainen, M.Liimatainen, J.Santaholma
**Kivelä Hospital, Helsinki**	A.Aimolahti, E.Huovinen
**Koskela Hospital, Helsinki**	V.Ilkka, M.Lehtimäki
**Kotka Health Center**	E.Pälikkö-Kontinen, A.Vanhanen
**Kouvola Health Center**	E.Koskinen, T.Siitonen
**Kuopio University Hospital**	E.Huttunen, R.Ikäheimo, P.Karhapää, P.Kekäläinen, M.Laakso, T.Lakka, E.Lampainen, L.Moilanen, S. Tanskanen, L.Niskanen, U.Tuovinen, I.Vauhkonen, E.Voutilainen
**Kuusamo Health Center**	T.Kääriäinen, E.Isopoussu
**Kuusankoski Hospital**	E.Kilkki, I.Koskinen, L.Riihelä
**Laakso Hospital, Helsinki**	T.Meriläinen, P.Poukka, R.Savolainen, N.Uhlenius
**Lahti City Hospital**	A.Mäkelä, M.Tanner
**Lapland Central Hospital, Rovaniemi**	L.Hyvärinen, K.Lampela, S.Pöykkö, T.Rompasaari, S.Severinkangas, T.Tulokas
**Lappeenranta Health Center**	P. Erola, L.Härkönen, P.Linkola, T.Pekkanen, I.Pulli, E.Repo
**Lohja Hospital**	T.Granlund, K.Hietanen, M.Porrassalmi, M.Saari, T.Salonen, M.Tiikkainen,
**Länsi-Uusimaa Hospital, Tammisaari**	I.-M.Jousmaa, J.Rinne
**Loimaa Health Center**	A.Mäkelä, P.Eloranta
**Malmi Hospital, Helsinki**	H.Lanki, S.Moilanen, M.Tilly-Kiesi
**Mikkeli Central Hospital**	A.Gynther, R.Manninen, P.Nironen, M.Salminen, T.Vänttinen
**Mänttä Regional Hospital**	I.Pirttiniemi, A-M.Hänninen
**North Karelian Hospital, Joensuu**	U-M.Henttula, P.Kekäläinen, M.Pietarinen, A.Rissanen, M.Voutilainen
**Nurmijärvi Health Center**	A.Burgos, K.Urtamo
**Oulaskangas Hospital, Oulainen**	E.Jokelainen, P-L.Jylkkä, E.Kaarlela, J.Vuolaspuro
**Oulu Health Center**	L.Hiltunen, R.Häkkinen, S.Keinänen-Kiukaanniemi
**Oulu University Hospital**	R.Ikäheimo
**Päijät-Häme Central Hospital**	H.Haapamäki, A.Helanterä, S.Hämäläinen, V.Ilvesmäki, H.Miettinen
**Palokka Health Center**	P.Sopanen, L.Welling
**Pieksämäki Hospital**	V.Sevtsenko, M.Tamminen
**Pietarsaari Hospital**	M-L.Holmbäck, B.Isomaa, L.Sarelin
**Pori City Hospital**	P.Ahonen, P.Merisalo, E.Muurinen, K.Sävelä
**Porvoo Hospital**	M.Kallio, B.Rask, S.Rämö
**Raahe Hospital**	A.Holma, M.Honkala, A.Tuomivaara, R.Vainionpää
**Rauma Hospital**	K.Laine, K.Saarinen, T.Salminen
**Riihimäki Hospital**	P.Aalto, E.Immonen, L.Juurinen
**Salo Hospital**	A.Alanko, J.Lapinleimu, P.Rautio, M.Virtanen
**Satakunta Central Hospital, Pori**	M.Asola, M.Juhola, P.Kunelius, M.-L.Lahdenmäki, P.Pääkkönen, M.Rautavirta

**Savonlinna Central Hospital**	T.Pulli, P.Sallinen, M.Taskinen, E.Tolvanen, T.Tuominen, H.Valtonen, A.Vartia, S-L.Viitanen
**Seinäjoki Central Hospital**	O.Antila, E.Korpi-Hyövälti, T.Latvala, E.Leijala, T.Leikkari, M.Punkari N.Rantamäki, H.Vähävuori
**South Karelia Central Hospital, Lappeenranta**	T.Ensala, E.Hussi, R.Härkönen, U.Nyholm, J.Toivanen
**Tampere Health Center**	A.Vaden, P.Alarotu, E.Kujansuu, H.Kirkkopelto-Jokinen, M.Helin, S.Gummerus, L.Calonius, T.Niskanen, T.Kaitala, T.Vatanen
**Tampere University Hospital**	P. Hannula, I.Ala-Houhala, R.Kannisto, T.Kuningas, P.Lampinen, M.Määttä,H.Oksala, T.Oksanen, A.Putila, H.Saha, K.Salonen, H.Tauriainen, S.Tulokas
**Tiirismaa Health Center, Hollola**	T.Kivelä, L.Petlin, L.Savolainen
**Turku Health Center**	A.Artukka, I.Hämäläinen, L.Lehtinen, E.Pyysalo, H.Virtamo, M.Viinikkala, M.Vähätalo
**Turku University Central Hospital**	K.Breitholz, R.Eskola, K.Metsärinne, U.Pietilä, P.Saarinen, R.Tuominen, S.Äyräpää
**Vaajakoski Health Center**	K.Mäkinen, P.Sopanen
**Valkeakoski Regional Hospital**	S.Ojanen, E.Valtonen, H.Ylönen, M.Rautiainen, T.Immonen
**Vammala Regional Hospital**	I.Isomäki, R.Kroneld, L.Mustaniemi, M.Tapiolinna-Mäkelä
**Vasa Central Hospital**	S.Bergkulla, U.Hautamäki, V-A.Myllyniemi, I.Rusk

Supplemental Table 2. Neuropsychological test variables.

		Description of the test	Variables	Cognitive function
**WAIS-IV Coding**[1]		A sequence of numbers, each paired with a corresponding hieroglyphic-like symbol. Using a key, the examinee writes the symbol corresponding to its number.	Number of correct responses in a 2-minute time limit	Processing speed
**Flexible Attention Test**[2,3]				
1	Reaction Time	24 light grey circles are randomly distributed on the touch screen. One circle at the time turns green. The task is to tap the green circle as quickly as possible.	Total time to complete the task (s)	Processing speed
2	Numbers	Numbers 1-24 are scattered in circles on the touch screen. The task is to tap the numbers in order from smallest to highest as quickly as possible.	Total time to complete the task (s)	Processing speed
3	Numbers and Letters	Numbers 1-12 and alphabets A-L are randomly distributed in circles on the touch screen. The task is to alternately tap numbers and letters in sequence of 1-A-2-B-3-C, etc.	Total time to complete the task (s) divided by number of correct responses (max. 24)	Executive functions (Cognitive flexibility)
4	Numbers and Shapes	Numbers 1-12 in circles and another set of numbers 1-12 in squares are randomly distributed on the touch screen. The task is to tap the numbers from smallest to largest, alternating between circles and squares.	Total time to complete the task (s) divided by number of correct responses (max. 12)	Executive functions (Cognitive flexibility)
5	Numbers and Months Forward	Numbers 1-12 and months Jan-Dec are randomly distributed in circles on the touch screen. The task is to alternately tap numbers and months in forward order in a sequence of 1-Jan-2-Feb-3-Mar, etc.	Total time to complete the task (s) divided by number of correct responses (max. 24)	Executive functions (Cognitive flexibility)
6	Numbers and Months Backward	Numbers 1-12 and months Jan-Dec are randomly distributed in circles on the touch screen. The task is to alternately tap numbers and months in backward order in a sequence of 1-Dec-2-Nov-3-Oct, etc.	Total time to complete the task (s) divided by number of correct responses (max. 24)	Executive functions (Cognitive flexibility)
7	Visuospatial Memory Span Forward	Corsi Block-Tapping type forward span task: 24 light grey circles are distributed on the screen, one at a time turning red. The task is to tap the same circles in the same order. The length of the sequence to be recalled gradually increases until the subject makes two consecutive errors in the same span length.	Maximum forward span score	Executive functions (Visuospatial working memory)
8	Visuospatial Memory Span Backward	Corsi Block-Tapping type backward span task: 24 light grey circles are distributed on the screen, one at a time turning red. The task is to tap the same circles, but in reverse order. The length of the sequence to be recalled gradually increases until the subject makes two consecutive errors in the same span length.	Maximum backward span score	Executive functions (Visuospatial working memory)
**Stroop Color-Naming**[4,5]		100 colours (written as XXXX) are printed in either red, green, blue, or yellow. The task is to name the colours as fast as possible.	Total time to complete the task (s)	Processing speed
**Stroop Color-Incongruent**[4,5]		100 coloured words are written so that no word for a colour matches the ink colour (e.g., the word "blue" printed in red ink). The task is to name the ink colour of the word as fast and precise as possible, while inhibiting the word meaning.	Total time to complete the task (s) divided by number of correct responses (max. 100)	Executive functions (Inhibition)

Supplemental Table 3. Cross-sectional associations of vascular density for superficial (SCP) and deep (DCP) retinal capillary plexus, and foveal avascular zone (FAZ) with cognitive tests of processing speed and executive function subdomains of inhibition, cognitive flexibility, and working memory.

	**Processing Speed**				**Executive Functions**						
					**Inhibition**	**Cognitive flexibility**				**Working memory**	
	**Coding**	**Stroop-II**	**FAT-RT**	**FAT-N**	**Stroop-III**	**FAT-NL**	**FAT-NS**	**FAT-NMF**	**FAT-NMB**	**FAT-MF**	**FAT-MB**
	**Standardized *β* (p-value)**										
SCP	**0.17 (0.031)**	**–0.19 (0.017)**†	**–0.26 (0.001)**†	**–0.17 (0.034)**	**–0.19 (0.018)**†	**–0.19 (0.018)**†	–0.15 (0.058)	**–0.26 (0.001)**†	**–0.17 (0.042)**	0.08 (0.311)	0.08 (0.356)
DCP	**0.22 (0.006)**†	**–0.23 (0.004)**†	**–0.21 (0.008)**†	–0.13 (0.118)	**–0.23 (0.004)**†	**–0.17 (0.036)**	–0.09 (0.259)	**–0.28 (<0.001)**†	–0.13 (0.116)	–0.07 (0.389)	0.09 (0.282)
FAZ	–0.02 (0.848)	0.16 (0.051)	0.10 (0.232)	0.08 (0.352)	0.03 (0.692)	**0.17 (0.034)**	0.02 (0.772)	0.06 (0.452)	0.04 (0.617)	0.01 (0.857)	**–0.29 (<0.001)**†

Univariate linear regression models with cognitive test scores as dependent variables. Independent variables included separately vascular density of SCP, vascular density of DCP, and FAZ in as univariate associations. Values are standardized beta coefficients (p-values). FAT, Flexible Attention Test; FAT-MB, FAT Visuospatial Memory Span Backward; FAT-MF, FAT Visuospatial Memory Span Forward; FAT-N, FAT Numbers; FAT-NL, FAT Numbers and Letters; FAT-NMB, FAT Numbers and Months Backward; FAT-NMF, FAT Numbers and Months Forward; FAT-NS, FAT Numbers and Shapes; FAT-RT, FAT Reaction Time; Stroop-II, Stroop Color-Naming; Stroop-III, Stroop Color-Incongruent. Associations that remain significant after false discovery rate correction are marked with †. Cohen's f^2 for significant associations between optical coherence tomography angiography markers and cognition ranged between 0.03 to 0.09. An illustration of FAT can be found in earlier study [3].

Supplemental Table 4. R^2 for univariate models and R^2 change for age beyond OCTA markers.

	Processing Speed				Executive Functions						
					Inhibition	Cognitive flexibility				Working memory	
	**Coding**	**Stroop-II**	**FAT-RT**	**FAT-N**	**Stroop-III**	**FAT-NL**	**FAT-NS**	**FAT-NMF**	**FAT-NMB**	**FAT-MF**	**FAT-MB**
**R^2 (SCP)**	0.03	0.04	0.07	0.03	0.04	0.04	0.02	0.07	0.03	0.01	0.01
**R^2 change (p)**	0.18 (<0.001)	0.04 (0.017)	0.22 (<0.001)	0.12 (<0.001)	0.07 (<0.001)	0.14 (<0.001)	0.26 (<0.001)	0.15 (<0.001)	0.15 (<0.001)	0.03 (0.025)	0.03 (0.054)
**R^2 (DCP)**	0.05	0.05	0.05	0.02	0.05	0.03	0.01	0.08	0.02	<0.01	0.01
**R^2 change (p)**	0.17 (<0.001)	0.03 (0.020)	0.24 (<0.001)	0.13 (<0.001)	0.07 (<0.001)	0.14 (<0.001)	0.28 (<0.001)	0.15 (<0.001)	0.16 (<0.001)	0.05 (0.007)	0.03 (0.069)
**R^2 (FAZ)**	<0.01	0.02	0.01	0.01	<0.01	0.03	<0.01	<0.01	<0.01	<0.01	0.08
**R^2 change (p)**	0.21 (<0.001)	0.05 (0.006)	0.27 (<0.001)	0.14 (<0.001)	0.09 (<0.001)	0.16 (<0.001)	0.28 (<0.001)	0.19 (<0.001)	0.17 (<0.001)	0.04 (0.014)	0.02 (0.069)

In the linear regression models, dependent variables were cognitive test scores, and independent variables included 1) in the univariate model the vascular density for superficial (SCP) and deep (DCP) retinal capillary plexus, and foveal avascular zone (FAZ), one at a time, and 2) in multivariable model additionally the age. FAT, Flexible Attention Test; FAT-MB, FAT Visuospatial Memory Span Backward; FAT-MF, FAT Visuospatial Memory Span Forward; FAT-N, FAT Numbers; FAT-NL, FAT Numbers and Letters; FAT-NMB, FAT Numbers and Months Backward; FAT-NMF, FAT Numbers and Months Forward; FAT-NS, FAT Numbers and Shapes; FAT-RT, FAT Reaction Time; Stroop-II, Stroop Color-Naming; Stroop-III, Stroop Color-Incongruent.

Supplemental Table 5. Cross-sectional associations of vascular density for superficial (SCP) retinal capillary plexus with cognitive tests of processing speed and executive function subdomains of inhibition, cognitive flexibility, and working memory, with adjustment of age, estimated glomerular filtration rate (eGFR) ml/min/1.73m², systolic blood pressure, and glycated haemoglobin (HbA_{1c}).

	**Processing Speed**				**Executive Functions**						
					**Inhibition**	**Cognitive flexibility**			**Working memory**		
	**Coding**	**Stroop-II**	**FAT-RT**	**FAT-N**	**Stroop-III**	**FAT-NL**	**FAT-NS**	**FAT-NMF**	**FAT-NMB**	**FAT-MF**	**FAT-MB**
	**Standardized *β* (p-value)**										
SCP	0.07 (0.345)	**-0.18 (0.031)**	-0.11 (0.126)	-0.06 (0.482)	-0.16 (0.062)	-0.10 (0.221)	-0.04 (0.609)	**-0.19 (0.013)**	-0.05 (0.548)	0.02 (0.855)	-0.03 (0.740)
Age	**-0.42 (<0.001)**	**0.25 (0.010)**	**0.46 (<0.001)**	**0.31 (0.001)**	**0.33 (<0.001)**	**0.42 (<0.001)**	**0.52 (<0.001)**	**0.45 (<0.001)**	**0.33 (<0.001)**	**-0.25 (0.011)**	-0.12 (0.247)
eGFR	-0.05 (0.525)	**0.19 (0.037)**	-0.03 (0.698)	-0.08 (0.392)	0.14 (0.111)	0.04 (0.673)	0.08 (0.331)	**0.17 (0.040)**	-0.04 (0.644)	0.01 (0.936)	0.19 (0.052)
SBP	-0.06 (0.483)	0.03 (0.742)	-0.02 (0.795)	0.02 (0.854)	0.00 (0.960)	-0.04 (0.610)	0.07 (0.380)	0.02 (0.795)	0.08 (0.330)	0.16 (0.070)	0.08 (0.416)
HbA_{1c}	-0.09 (0.216)	0.07 (0.390)	**0.17 (0.016)**	0.09 (0.267)	-0.01 (0.938)	0.00 (0.962)	0.06 (0.365)	0.08 (0.306)	0.13 (0.093)	-0.07 (0.423)	-0.15 (0.092)

Multivariable linear regression models with cognitive test scores as dependent variables. Independent variables included vascular density of SCP and additionally age, eGFR ml/min/1.73m², systolic blood pressure, and HbA_{1c}. Values are standardized beta coefficients (p-values). FAT, Flexible Attention Test; FAT-MB, FAT Visuospatial Memory Span Backward; FAT-MF, FAT Visuospatial Memory Span Forward; FAT-N, FAT Numbers; FAT-NL, FAT Numbers and Letters; FAT-NMB, FAT Numbers and Months Backward; FAT-NMF, FAT Numbers and Months Forward; FAT-NS, FAT Numbers and Shapes; FAT-RT, FAT Reaction Time; Stroop-II, Stroop Color-Naming; Stroop-III, Stroop Color-Incongruent; SBP, systolic blood pressure. Associations for SCP and cognition did not remain significant after false discovery rate correction. The independent contribution of SCP on cognition was small (Cohen's f^2 for significant associations ranged between 0.03 to 0.04). An illustration of FAT can be found in earlier study [3].

Supplemental Table 6. Cross-sectional associations of vascular density for deep (DCP) retinal capillary plexus with cognitive tests of processing speed and executive function subdomains of inhibition, cognitive flexibility, and working memory, with adjustment of age, estimated glomerular filtration rate (eGFR) ml/min/1.73m², systolic blood pressure, and glycated haemoglobin (HbA_{1c}).

	**Processing Speed**				**Executive Functions**						
					**Inhibition**	**Cognitive flexibility**				**Working memory**	
	**Coding**	**Stroop-II**	**FAT-RT**	**FAT-N**	**Stroop-III**	**FAT-NL**	**FAT-NS**	**FAT-NMF**	**FAT-NMB**	**FAT-MF**	**FAT-MB**
	**Standardized β (p-value)**										
DCP	0.13 (0.081)	**-0.21 (0.012)**	-0.08 (0.243)	-0.03 (0.680)	**-0.19 (0.021)**	-0.08 (0.327)	0.02 (0.763)	**-0.21 (0.006)**	-0.03 (0.688)	-0.15 (0.077)	0.02 (0.843)
Age	**-0.41 (<0.001)**	**0.23 (0.017)**	**0.46 (<0.001)**	**0.31 (0.001)**	**0.31 (0.001)**	**0.42 (<0.001)**	**0.53 (<0.001)**	**0.43 (<0.001)**	**0.33 (<0.001)**	**-0.29 (0.004)**	-0.11 (0.288)
eGFR	-0.05 (0.542)	0.17 (0.060)	-0.05 (0.496)	-0.09 (0.305)	0.12 (0.156)	0.02 (0.831)	0.06 (0.410)	0.14 (0.076)	-0.05 (0.552)	0.03 (0.731)	0.18 (0.059)
SBP	-0.07 (0.404)	0.04 (0.672)	-0.02 (0.784)	0.01 (0.874)	0.01 (0.882)	-0.04 (0.607)	0.06 (0.437)	0.03 (0.726)	0.08 (0.337)	0.19 (0.038)	0.07 (0.451)
HbA_{1c}	-0.09 (0.207)	0.08 (0.327)	**0.18 (0.011)**	0.09 (0.240)	0.00 (0.993)	0.00 (0.966)	0.07 (0.321)	0.09 (0.242)	0.13 (0.083)	-0.08 (0.351)	-0.14 (0.103)

Multivariable linear regression models with cognitive test scores as dependent variables. Independent variables included vascular density of DCP and additionally age, eGFR ml/min/1.73m², systolic blood pressure, and HbA_{1c}. Values are standardized beta coefficients (p-values). FAT, Flexible Attention Test; FAT-MB, FAT Visuospatial Memory Span Backward; FAT-MF, FAT Visuospatial Memory Span Forward; FAT-N, FAT Numbers; FAT-NL, FAT Numbers and Letters; FAT-NMB, FAT Numbers and Months Backward; FAT-NMF, FAT Numbers and Months Forward; FAT-NS, FAT Numbers and Shapes; FAT-RT, FAT Reaction Time; Stroop-II, Stroop Color-Naming; Stroop-III, Stroop Color-Incongruent; SBP, systolic blood pressure. Associations for DCP and cognition did not remain significant after false discovery rate correction. The independent contribution of DCP on cognition was small (Cohen's f^2 for significant associations ranged between 0.02 to 0.05). An llustration of FAT can be found in earlier study [3].

Supplemental Table 7. Cross-sectional associations of foveal avascular zone (FAZ) with cognitive tests of processing speed and executive function subdomains of inhibition, cognitive flexibility, and working memory, with adjustment of age, estimated glomerular filtration rate (eGFR) ml/min/1.73m², systolic blood pressure, and glycated haemoglobin (HbA_{1c}).

	**Processing Speed**				**Executive Functions**						
					**Inhibition**	**Cognitive flexibility**				**Working memory**	
	**Coding**	**Stroop-II**	**FAT-RT**	**FAT-N**	**Stroop-III**	**FAT-NL**	**FAT-NS**	**FAT-NMF**	**FAT-NMB**	**FAT-MF**	**FAT-MB**
	**Standardized β (p-value)**										
FAZ	0.02 (0.765)	0.14 (0.074)	0.04 (0.609)	0.04 (0.648)	0.01 (0.893)	0.14 (0.074)	-0.02 (0.765)	0.02 (0.767)	0.00 (0.998)	0.05 (0.561)	**-0.25 (0.003)**†
Age	**-0.44 (<0.001)**	**0.26 (0.006)**	**0.48 (<0.001)**	**0.31 (<0.001)**	**0.35 (<0.001)**	**0.42 (<0.001)**	**0.53 (<0.001)**	**0.48 (<0.001)**	**0.34 (<0.001)**	**-0.26 (0.009)**	-0.09 (0.354)
eGFR	-0.03 (0.703)	0.15 (0.096)	-0.06 (0.426)	-0.09 (0.291)	0.10 (0.257)	0.02 (0.843)	0.07 (0.396)	0.12 (0.152)	-0.05 (0.517)	0.01 (0.870)	0.16 (0.083)
SBP	-0.05 (0.576)	0.02 (0.806)	-0.03 (0.698)	0.01 (0.884)	-0.01 (0.874)	-0.04 (0.625)	0.06 (0.426)	0.00 (0.999)	0.08 (0.362)	0.17 (0.059)	0.05 (0.598)
HbA_{1c}	-0.10 (0.167)	0.08 (0.328)	**0.18 (0.011)**	0.09 (0.245)	0.01 (0.887)	0.00 (0.965)	0.07 (0.319)	0.10 (0.200)	0.13 (0.079)	-0.07 (0.384)	-0.12 (0.167)

Multivariable linear regression models with cognitive test scores as dependent variables. Independent variables included area of FAZ and additionally age, eGFR ml/min/1.73m², systolic blood pressure, and HbA_{1c}. Values are standardized beta coefficients (p-values). FAT, Flexible Attention Test; FAT-MB, FAT Visuospatial Memory Span Backward; FAT-MF, FAT Visuospatial Memory Span Forward; FAT-N, FAT Numbers; FAT-NL, FAT Numbers and Letters; FAT-NMB, FAT Numbers and Months Backward; FAT-NMF, FAT Numbers and Months Forward; FAT-NS, FAT Numbers and Shapes; FAT-RT, FAT Reaction Time; Stroop-II, Stroop Color-Naming; Stroop-III, Stroop Color-Incongruent; SBP, systolic blood pressure. Association between FAZ and FAT-MB remained significant after false discovery rate correction, which is indicated with †. The independent contribution of FAZ on FAT-MB was small (Cohen's f^2=0.07). An illustration of FAT can be found in earlier study [3].

Supplemental Table 8. Cross-sectional associations of vascular density for superficial (SCP) retinal capillary plexus with cognitive tests of processing speed and executive function subdomains of inhibition, cognitive flexibility, and working memory, with adjustment of age, education, depression, and smoking.

	**Processing Speed**				**Executive Functions**						
					**Inhibition**	**Cognitive flexibility**			**Working memory**		
	**Coding**	**Stroop-II**	**FAT-RT**	**FAT-N**	**Stroop-III**	**FAT-NL**	**FAT-NS**	**FAT-NMF**	**FAT-NMB**	**FAT-MF**	**FAT-MB**
	**Standardized β (p-value)**										
SCP	0.08 (0.311)	**-0.17 (0.048)**	-0.14 (0.056)	-0.08 (0.349)	-0.15 (0.066)	-0.10 (0.197)	-0.04 (0.561)	**-0.17 (0.029)**	-0.08 (0.285)	0.06 (0.485)	0.02 (0.832)
Age	**-0.42 (<0.001)**	**0.17 (0.049)**	**0.48 (<0.001)**	**0.36 (<0.001)**	**0.24 (0.004)**	**0.37 (<0.001)**	**0.49 (<0.001)**	**0.38 (<0.001)**	**0.37 (<0.001)**	-0.15 (0.073)	-0.17 (0.059)
Education	0.06 (0.44)	-0.06 (0.457)	-0.04 (0.614)	-0.04 (0.627)	-0.09 (0.284)	-0.04 (0.620)	-0.13 (0.068)	-0.13 (0.076)	-0.12 (0.135)	0.16 (0.052)	0.13 (0.137)
Depression	0.05 (0.801)	-0.01 (0.964)	-0.17 (0.316)	-0.15 (0.410)	-0.03 (0.879)	-0.15 (0.412)	-0.22 (0.186)	-0.07 (0.689)	0.02 (0.901)	**0.47 (0.015)**	0.35 (0.095)
Smoking	0.08 (0.631)	0.12 (0.464)	-0.11 (0.456)	0.03 (0.853)	0.00 (0.982)	0.00 (0.989)	**0.32 (0.033)**	0.06 (0.690)	0.16 (0.331)	-0.12 (0.462)	0.03 (0.850)

Multivariable linear regression models with cognitive test scores as dependent variables. Independent variables included vascular density of SCP and additionally age, years of education, smoking status (current or former smoker/never smoked), and history of depression (yes/no). Values are standardized beta coefficients (p-values). FAT, Flexible Attention Test; FAT-MB, FAT Visuospatial Memory Span Backward; FAT-MF, FAT Visuospatial Memory Span Forward; FAT-N, FAT Numbers; FAT-NL, FAT Numbers and Letters; FAT-NMB, FAT Numbers and Months Backward; FAT-NMF, FAT Numbers and Months Forward; FAT-NS, FAT Numbers and Shapes; FAT-RT, FAT Reaction Time; Stroop-II, Stroop Color-Naming; Stroop-III, Stroop Color-Incongruent; SBP, systolic blood pressure. The independent contribution of SCP on cognition was small (Cohen's f^2 for significant association between SCP and Stroop-II was 0.03). Associations for SCP and cognition did not remain significant after false discovery rate correction. An illustration of FAT can be found in earlier study [3].

Supplemental Table 9. Cross-sectional associations of vascular density for deep (DCP) retinal capillary plexus with cognitive tests of processing speed and executive function subdomains of inhibition, cognitive flexibility, and working memory, with adjustment of age, education, depression, and smoking.

	**Processing Speed**				**Executive Functions**						
					**Inhibition**	**Cognitive flexibility**				**Working memory**	
	**Coding**	**Stroop-II**	**FAT-RT**	**FAT-N**	**Stroop-III**	**FAT-NL**	**FAT-NS**	**FAT-NMF**	**FAT-NMB**	**FAT-MF**	**FAT-MB**
	**Standardized β (p-value)**										
DCP	0.14 (0.071)	**-0.20 (0.014)**	-0.10 (0.158)	-0.03 (0.675)	**-0.20 (0.014)**	-0.08 (0.278)	0.03 (0.641)	**-0.20 (0.010)**	-0.04 (0.596)	-0.12 (0.142)	0.03 (0.718)
Age	**-0.41 (<0.001)**	0.16 (0.050)	**0.50 (<0.001)**	**0.37 (<0.001)**	**0.23 (0.004)**	**0.38 (<0.001)**	**0.51 (<0.001)**	**0.38 (<0.001)**	**0.38 (<0.001)**	**-0.20 (0.018)**	-0.17 (0.065)
Education	0.05 (0.482)	-0.05 (0.525)	-0.03 (0.670)	-0.04 (0.648)	-0.08 (0.331)	-0.03 (0.660)	-0.13 (0.068)	-0.13 (0.095)	-0.11 (0.145)	**0.16 (0.047)**	0.13 (0.142)
Depression	0.03 (0.845)	0.00 (0.984)	-0.17 (0.330)	-0.15 (0.411)	-0.02 (0.931)	-0.15 (0.425)	-0.23 (0.176)	-0.06 (0.738)	0.02 (0.900)	**0.49 (0.011)**	0.35 (0.095)
Smoking	0.09 (0.549)	0.09 (0.596)	-0.14 (0.354)	0.02 (0.921)	-0.04 (0.827)	-0.02 (0.911)	**0.31 (0.036)**	0.03 (0.859)	0.14 (0.382)	-0.11 (0.493)	0.04 (0.832)

Multivariable linear regression models with cognitive test scores as dependent variables. Independent variables included vascular density of DCP and additionally age, years of education, smoking status (current or former smoker/never smoked), and history of depression (yes/no). Values are standardized beta coefficients (p-values). FAT, Flexible Attention Test; FAT-MB, FAT Visuospatial Memory Span Backward; FAT-MF, FAT Visuospatial Memory Span Forward; FAT-N, FAT Numbers; FAT-NL, FAT Numbers and Letters; FAT-NMB, FAT Numbers and Months Backward; FAT-NMF, FAT Numbers and Months Forward; FAT-NS, FAT Numbers and Shapes; FAT-RT, FAT Reaction Time; Stroop-II, Stroop Color-Naming; Stroop-III, Stroop Color-Incongruent; SBP, systolic blood pressure. The independent contribution of DCP on cognition was small (Cohen's f^2 for significant associations ranged between 0.04 to 0.05). Associations for DCP and cognition did not remain significant after false discovery rate correction. An illustration of FAT can be found in earlier study [3].

Supplemental Table 10. Cross-sectional associations of foveal avascular zone (FAZ) with cognitive tests of processing speed and executive function subdomains of inhibition, cognitive flexibility, and working memory, with adjustment of age, education, depression, and smoking.

	Processing Speed				Executive Functions						
					Inhibition	Cognitive flexibility				Working memory	
	Coding	Stroop-II	FAT-RT	FAT-N	Stroop-III	FAT-NL	FAT-NS	FAT-NMF	FAT-NMB	FAT-MF	FAT-MB
	Standardized β (p-value)										
FAZ	0.03 (0.728)	0.15 (0.065)	0.04 (0.606)	0.03 (0.720)	0.01 (0.913)	0.13 (0.086)	-0.04 (0.594)	0.01 (0.894)	0.01 (0.920)	0.07 (0.380)	**-0.23 (0.005)**
Age	**-0.45 (<0.001)**	**0.20 (0.015)**	**0.52 (<0.001)**	**0.38 (<0.001)**	**0.28 (<0.001)**	**0.39 (<0.001)**	**0.51 (<0.001)**	**0.43 (<0.001)**	**0.39 (<0.001)**	**-0.17 (0.032)**	-0.15 (0.078)
Education	0.06 (0.436)	-0.04 (0.600)	-0.03 (0.679)	-0.03 (0.666)	-0.08 (0.313)	-0.02 (0.763)	-0.13 (0.063)	-0.13 (0.091)	-0.11 (0.145)	**0.16 (0.044)**	0.10 (0.241)
Depression	0.06 (0.737)	0.05 (0.794)	-0.16 (0.360)	-0.15 (0.448)	-0.03 (0.865)	-0.10 (0.607)	-0.24 (0.159)	-0.07 (0.685)	0.02 (0.904)	**0.51 (0.010)**	0.25 (0.228)
Smoking	0.09 (0.553)	0.11 (0.508)	-0.13 (0.383)	0.02 (0.901)	-0.03 (0.856)	0.00 (0.995)	**0.31 (0.039)**	0.03 (0.830)	0.14 (0.375)	-0.10 (0.538)	0.02 (0.932)

Multivariable linear regression models with cognitive test scores as dependent variables. Independent variables included area of FAZ and additionally age, years of education, smoking status (current or former smoker/never smoked), and history of depression (yes/no). Values are standardized beta coefficients (p-values). FAT, Flexible Attention Test; FAT-MB, FAT Visuospatial Memory Span Backward; FAT-MF, FAT Visuospatial Memory Span Forward; FAT-N, FAT Numbers; FAT-NL, FAT Numbers and Letters; FAT-NMB, FAT Numbers and Months Backward; FAT-NMF, FAT Numbers and Months Forward; FAT-NS, FAT Numbers and Shapes; FAT-RT, FAT Reaction Time; Stroop-II, Stroop Color-Naming; Stroop-III, Stroop Color-Incongruent; SBP, systolic blood pressure. The independent contribution of FAZ on FAT-MB was very small (Cohen's f^2 <0.01). Association for FAZ and FAT-MB did not remain significant after false discovery rate correction. An illustration of FAT can be found in earlier study [3].

References

1. Wechsler D. *Wechsler Adult Intelligence Scale (WAIS-IV)*. 4th edition. TX: Psychological Corporation; 2008 [Finnish version published 2012].

2. Tikkanen V, Krüger J, Heikkinen AL, et al. A Novel Computerized Flexible Attention Test in Detecting Executive Dysfunction of Patients with Early-Onset Cognitive Impairment and Dementia. *Arch Clin Neuropsychol*. 2024;39(7):817-828. doi:10.1093/arclin/acae026

3. Jokinen H, Laakso HM, Arola A, et al. Executive functions and processing speed in covert cerebral small vessel disease. *Eur J Neurol*. 2025;31(1):e16533. doi:10.1111/ene.16533

4. MacLeod CM. Half a century of research on the Stroop effect: An integrative review. *Psychol Bull*. 1991;109(2):163-203. doi:10.1037/0033-2909.109.2.163

5. Golden CJ. Stroop Colour Word Test. In: *A Manual for Clinical and Experimental Uses*. Stoelting; 1978.
